# Cranial shape during early neonatal period in Japanese newborns: Observational study

**DOI:** 10.1097/MD.0000000000043473

**Published:** 2025-07-18

**Authors:** Yukari Tanaka, Hiroshi Miyabayashi, Nobuhiko Nagano, Yuri Kubota, Chihiro Mukai, Aya Nakanomori, Koichiro Hara, Katsuya Saito, Risa Kato, Ichiro Morioka

**Affiliations:** a Department of Pediatrics and Child Health, Nihon University School of Medicine, Tokyo, Japan; b Department of Pediatrics, Kasukabe Medical Center, Saitama, Japan.

**Keywords:** brachycephaly, cranial shape, newborn, plagiocephaly, stereophotogrammetry

## Abstract

This study aimed to investigate whether cranial shape measurements obtained immediately after birth can be used to determine the timeline and mechanisms underlying the development and progression of cranial deformities in healthy infants. This study examined the cranial geometry of normal newborns immediately after birth at Nihon University Itabashi Hospital and Kasukabe Medical Center. Measurements were obtained using stereophotogrammetry and 3-dimensional cranial data were analyzed using image analysis software. According to international criteria, positional deformational plagiocephaly (PDP) was identified at a cranial vault asymmetry index >3.5%, whereas positional brachycephaly was identified at a cephalic index ≥81%. These data were compared with those from a previously reported database of 1-month-old infants. A total of 130 newborns with a mean gestational age of 39 weeks, a mean birth weight of 3075 g, a mean head circumference of 33.5 cm, and a male sex ratio of 48.5% were included. The mean age at measurement was 3.3 days. The prevalence of PDP and positional brachycephaly was 19.2% and 86.2%, respectively. No notable differences in the background characteristics were observed between the PDP and non-PDP groups. The mean cephalic index was significantly lower in breech fetuses (*P* = .015), with no significant differences between the delivery methods. The symmetry-related parameters of cranial deformities were more pronounced at 1 month than at birth, with a substantially higher median cranial vault asymmetry index of 4.9% and 2.1%, respectively (*P* < .01). The prevalence of PDP increased significantly from 19.2% at birth to 66.1% at 1 month of age. Our findings suggest that cranial deformities become more pronounced within the first month after birth. Prevention at an early stage will be the focus of future research.

## 1. Introduction

Because the maximum cranial diameter of a full-term fetus is larger than that of a bony birth canal, the bones forming the cranial crown are not fully ossified at birth. Consequently, the cranial sutures and fenestrae remain open and easily deformed. Common cranial deformities observed immediately after birth include head lengthening, caput succedaneum, and bone intussusception, all of which typically improve within the first few days after birth.

Despite the termination of substantial environmental changes associated with birth, the newborn’s cranial shape can be easily and passively changed by external influences such as gravity, positioning, and sleeping posture.^[1]^ Furthermore, the American Academy of Pediatrics’ 1992 “Back to Sleep” campaign, which recommended placing infants in the supine position to reduce the risk of sudden infant death syndrome, substantially increased the incidence of positional deformational plagiocephaly (PDP) in the United States.^[2]^ The prevalence of PDP in infants has increased recently, leading to the implementation of helmet therapy.^[3,4]^

Numerous studies of the posttreatment course of cranial geometry indicated that treatment for severe PDP should begin at 6 months of age.^[5]^ However, the timeline and mechanisms underlying cranial deformity development and progression in healthy infants remain unclear. Previous reports have indicated that the prevalence of PDP is 64.7% at 1 month of age,^[6]^ peaks at 3 months of age, and improves by 6 months of age.^[7,8]^ In this study, we measured the newborn cranial shape during the early neonatal period and compared cranial deformation with that of previously reported healthy 1-month-old infants to elucidate the timing at which it begins.

## 2. Materials and methods

### 2.1. Ethics

This study was conducted at Nihon University Itabashi Hospital and Kasukabe Medical Center. The study protocol was approved by the local ethics committee of Nihon University Itabashi Hospital (approval no. RK-221011-1; Kasukabe Medical Center approval no. KMC C2022-044). Written informed consent was obtained from the parents of all participants.

### 2.2. Target cases

This study included full-term newborns born at Nihon University Itabashi Hospital and Kasukabe Medical Center between January 2023 and August 2024. All participants were born to Japanese parents at ≥37 weeks of gestation. Newborns with neonatal asphyxia and a 5-minute Apgar score ≤7 were excluded. Pediatricians examined the newborns ≥24 hours after birth. Patients with cephalohematoma, bone intussusception, or subgaleal hemorrhage were excluded because these conditions were considered to affect cranial shape. The pediatrician registered newborns who were determined to have healed from birth-related deformities (caput succedaneum).

Data from healthy Japanese 1-month-old infants who participated in a previous follow-up study were used for comparison.^[6–8]^

### 2.3. Data acquisition and analysis

#### 2.3.1. Measurement technique

When the newborns were lying in bed, their heads were covered with an elastic wig cap to minimize the effects of hair. Stereophotogrammetry (VECTRA H2; Canfield Scientific, Parsippany-Troy Hills) was conducted at 9 different angles (front, back, left, right, 4 oblique angles, and overhead) to capture 3-dimensional data converted into STL files in standard 3D format images.^[9]^ The images were analyzed using a custom program developed by Berry Inc., (Tokyo, Japan). This measurement method has been widely used in Japan. Inter- and intrarater reliability indices in our facility have been reported, and we found no reproducibility problems in comparison with human measurements.^[10]^

**Figure 1.**
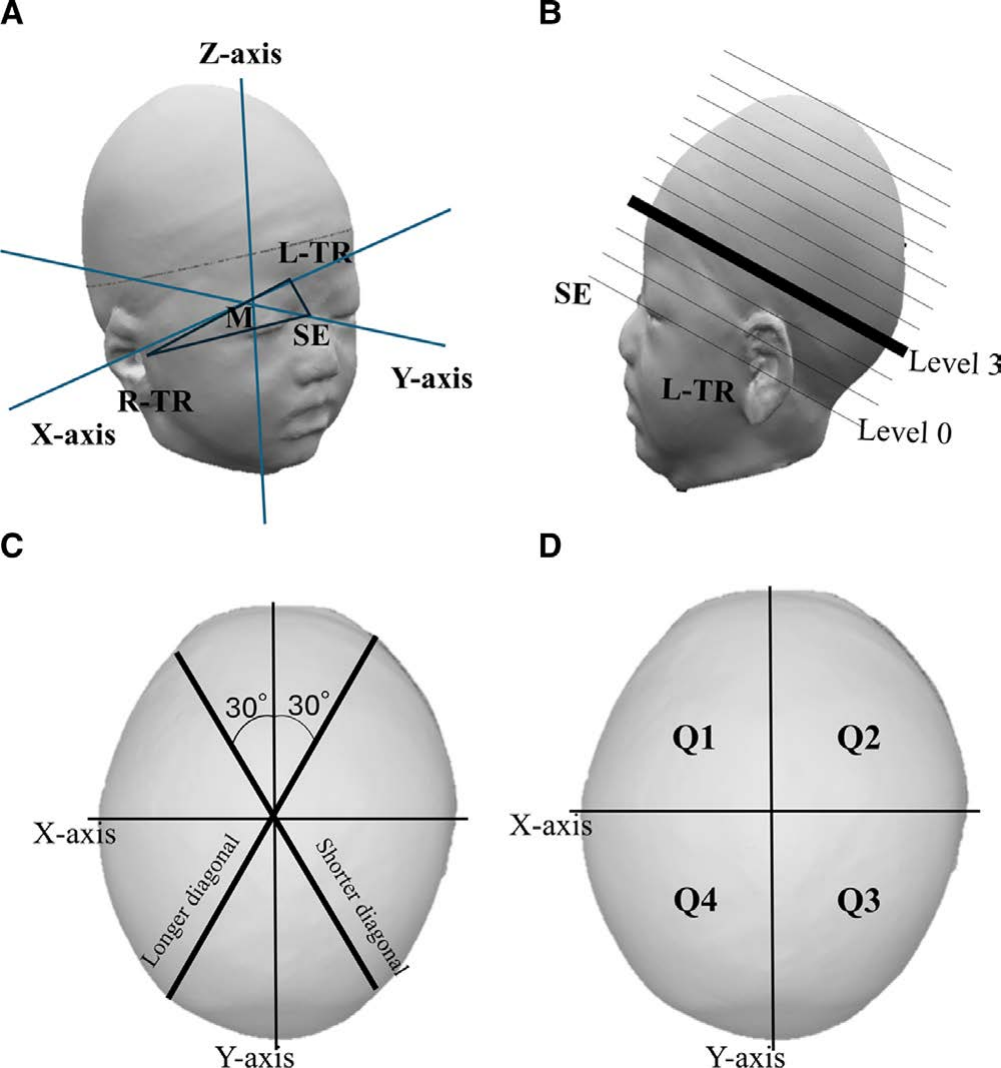
Three-dimensional images. (A) Illustration showing how the reference plane (level 0) and the *X*- and *Y*-axes were determined. (B) Illustration showing how the plane and *Z*-axis for levels 0 to 10 are determined. (C) Cross-sectional view of the level 3 measurement plane. The *X*-axis, *Y*-axis, and 30° diagonals were used to quantify cranial asymmetry and the cranial vault asymmetry index. (D) Methods used to determine anterior and posterior symmetry ratios. L-TR = left tragion, M = midpoint between tragions, Q = quadrant, R-TR = right tragion, SE = lion, TR = tragion.

Three landmarks (sellion and left and right tragions) were established on the 3D cranial images.^[11,12]^ The sellion was the lowest point of the nasal root, and the tragion was the upper margin of the tragus. The setting points were not changed for newborns. The plane connecting these 3 points served as the reference plane (level 0), and the *X*- and *Y*-axes were defined using analysis software (Fig. 1A).^[11,13]^ Ten equally spaced parallel sections were created from level 0 to the top of the head (Fig. 1B).^[11,12]^

#### 2.3.2. Growth-related parameters

Cranial volume (CrV) is the volume between levels 2 and 8.^[11,12]^ Cranial height was defined as the *Z*-axis length from level 0 to the top of the head.^[14]^ The cross-section at level 3 served as the measurement plane.^[11,12]^ Figure 1C shows a cross-sectional view of the measurement plane. The cranial length (CrL) and cranial width (CrW) were measured as the lengths of the measurement plane in the *Y*- and *X*-axis directions, respectively. Cranial circumference (CC) was defined as the circumference of the measurement surface.

#### 2.3.3. Symmetry-related parameters

The cephalic index (CI) was calculated as CrW/CrL (%).^[15]^ Cranial asymmetry (CA) was defined as the difference between the lengths of the longer and shorter axes 30° from the *y*-axis of the measurement plane on the left and right sides, respectively. The cranial vault asymmetry index (CVAI) is defined as the CA/shorter axis length (%).^[16,17]^

The 4 regions of the head divided by the *X*- and *Y*-axis planes were designated as the left frontal (Q1), right frontal (Q2), right occipital (Q3), and left occipital (Q4) quadrants (Fig. 1D). The anterior symmetry rate (ASR) was defined as the ratio of the smaller volume to the larger volume in Q1 and Q2 (Q1/Q2 or Q2/Q1, in which the denominator was larger). Similarly, the posterior symmetry rate (PSR) was defined as the volume ratio in the occipital region (Q3/Q4 or Q4/Q3).^[3,11]^ According to international criteria, PDP was defined as a CVAI of >3.5%^[16]^ and positional brachycephaly (PBC) as a CI of ≥81%.^[14]^

### 3.1. Statistical analyses

#### 3.1.1. Study 1: Cranial shape of newborns

The mean and standard deviation of each parameter were calculated. The Shapiro–Wilk test was used to test for normality. For parameters with a non-normal distribution (*P* < .05), the median and interquartile ranges were appended. Newborns were divided into those with and without PDP, defined as a CVAI > 3.5%. Univariate analysis was conducted to examine the risk factors for substantial differences in background characteristics, including the number of pregnancies, fetal position, delivery method, gestational age, head circumference at birth, and sex. The CI values were compared based on the delivery method and fetal position.

#### 3.1.2. Study 2: Comparison with 1-month-old infants

Symmetry-related parameters were compared with previously reported cranial shape data from 1-month-old healthy infants.^[6,8]^ EZR (version 1.68) was used for statistical analyses.^[18]^ The univariate analysis was performed using Student *t* test, Mann–Whitney *U* test, chi-squared test, and post hoc power was also considered for each statistic.

## 4. Results

### 4.1. Participants’ background characteristics

The participants’ background characteristics are presented in Table 1. A total of 130 newborns were included in this study. No cases were excluded because of cephalohematoma or other reasons. The mean age of the participants at the time of measurement was 3.3 days. The mean age of the participants in the 1-month-old group was 35.7 days. No considerable differences were observed in the background characteristics.

**Table 1:** Characteristics of participants.

	0-mo-old	1-mo-old	*P*-value	Power
Number	130	165		
Maternal age, yr	32.8 ± 4.8	33.1 ± 5.2	.54	0.07
First birth	77 (59.2)	80 (48.5)	.08	0.40
Delivery method			.13	0.32
Vaginal	65 (50.0)	98 (59.4)		
Cesarean section	65 (50.0)	67 (40.6)		
Fetal presentation cephalic	122 (93.8)	151 (91.5)	.51	0.06
Gestational age, wk	39 ± 1.3	39 ± 1.3	.49	0.10
Birth weight, g	3075 ± 381	3048 ± 367	.54	0.09
Birth head circumference, cm	33.5 ± 1.2	33.8 ± 1.4	.60	0.50
Male	63 (48.5)	89 (53.9)	.41	0.12
Measured day age	3.3 ± 2.0	35.7 ± 6.3	<.01	1.00

Data are presented as the mean value ± standard deviation or number (%).

### 4.2. Study 1: Newborn cranial shape

Table 2 lists the mean values of these parameters. Parameters that exhibited a non-normal distribution (CA, CAVI, ASR, and PSR) were appended along with their medians and interquartile ranges. PDP was observed in 25 newborns (19.2%) and PBC in 112 newborns (86.2%). Infants of different sexes showed differences in growth-related parameters; however, no notable differences were found in symmetry-related parameters or the prevalence of PDP.

**Table 2:** Cranial shape of newborns.

	Whole	Male	Female	*P* value
Number	130	63	67	
Measured day age	3.3 ± 2.0	2.9 ± 1.8	3.6 ± 2.0	.06
Cranial length (mm)	117 ± 4.5	118 ± 4.5	117 ± 4.4	.08
Cranial width (mm)	98 ± 3.5	100 ± 2.9	97 ± 3.6	<.01
Cranial height (mm)	89 ± 3.7	90 ± 3.1	88 ± 4.0	<.01
Cranial circumference (mm)	341 ± 11	344 ± 10	338 ± 11	<.01
Cranial volume (mL)	428 ± 39	439 ± 35	417 ± 40	<.01
Cephalic index (%)	84.0 ± 3.0	84.5 ± 2.8	83.5 ± 3.0	.08
Cranial asymmetry (mm)	2.5 ± 1.7	2.5 ± 1.8	2.5 ± 1.7	.86
	2.3 (1.0–3.5)	2.3 (1.0–3.5)	2.3 (1.1–3.4)	
Cranial vault asymmetry index (%)	2.2 ± 1.6	2.2 ± 1.6	2.2 ± 1.6	.94
	2.1 (0.9–3.2)	2.1 (0.9–3.3)	2.2 (1.0–3.0)	
Anterior symmetry rate (%)	94.1 ± 4.3	94.5 ± 4.2	93.8 ± 4.4	.39
	94.6 (91.4–97.6)	94.8 (91.9–98.1)	94.0 (91.3–97.3)	
Posterior symmetry rate (%)	93.8 ± 5.1	94.1 ± 5.0	93.5 ± 5.2	.56
	95.2 (91.8–97.5)	95.4 (92.1–97.6)	95.0 (90.8–97.5)	
Positional deformational plagiocephaly; number (%)	25 (19.2)	13 (20.6)	12 (17.9)	.84
Positional brachycephaly; number (%)	112 (86.2)	57 (90..5)	55 (82.1)	.21

Data are presented as the mean value ± standard deviation or number (%).

Parameters that exhibited non-normal distribution were appended to their medians and interquartile ranges (cranial asymmetry, cranial vault asymmetry index, anterior symmetry rate, and posterior symmetry rate).

Table 3 shows a comparison between the PDP and normal groups. No notable differences were observed in the number of pregnancies, fetal position, method of delivery, gestational weeks at birth, head circumference at birth, or sex.

**Table 3:** Compares the positional deformational plagiocephaly and normal groups background.

	Positional deformational plagiocephaly	Non-positional deformational plagiocephaly	*P*-value	Power
Number	25	105		
First birth	14 (56.0)	63 (60.0)	.82	
Delivery method			.66	0.06
Vaginal	11 (44.0)	54 (51.4)		
Cesarean section	14 (56.0)	51 (48.6)		
Fetal presentation cephalic	23 (92.0)	99 (94.3)	.65	0.31
Gestational age, wk	39 ± 1.3	39 ± 1.2	.40	0.06
Birth weight, g	3061 ± 380	3133 ± 389	.40	0.13
Birth head circumference, cm	33.5 ± 1.2	33.7 ± 1.4	.36	0.10
Male	13 (52.0)	50 (47.6)	.82	
Measured day age	3.2 ± 1.8	3.6 ± 2.3	.26	0.14

Data are presented as the mean value ± standard deviation or number (%).

CI was compared between the vaginal delivery and cesarean section groups. Both groups had the same number of participants (N = 65). The CI for the vaginal delivery group was 84.5 ± 3.1% (mean value ± standard deviation), whereas the CI for cesarean section was 83.5 ± 2.1%, which was not significantly different (*P* = .055, detection power 0.50). The CI was also compared between the cephalic and breech position groups. The number of participants in the cephalic position group was 122, whereas that in the breech position group only included 8. The CI for the cephalic position group was 84.1 ± 2.9%, whereas that for the breech position group was 81.5 ± 2.3% (*P* = .015, detection power 0.71).

### 4.3. Study 2: Comparison with 1-month-old infants

Table 4 compares the symmetry-related parameters between the newborn and 1-month-old groups. All parameters were considerably worse, and the prevalence of PDP was notably higher in the 1-month-old group. The detection power of symmetry-related parameters, such as CA, was sufficient. However, the detection rate was low only in ASR and CI. The prevalence of PDP was significantly higher at 1 month, with a high detection power, but that of PBC did not change significantly and had weak detection power.

**Table 4:** Comparison of symmetry-related parameters between the 0-mo-old and 1-mo-old groups.

	0-mo-old	1-mo-old	*P*-value	Power
Number	130	165		
Measured day age (d)	3.3 ± 2.0	35.7 ± 6.3	<.01	1.0
Cranial asymmetry (mm)	2.3 (1.0–3.5)	6.1 (3.5–8.5)	<.01	1.0
Cranial vault asymmetry index (%)	2.1 (0.9–3.2)	4.9 (2.8–6.8)	<.01	1.0
Anterior symmetry rate (%)	94.6 (91.4–97.6)	93.8 (89.8–96.6)	.03	0.62
Posterior symmetry rate (%)	95.2 (91.8–97.5)	92.2 (85.6–96.9)	<.01	0.95
Cephalic index (%)	84.0 ± 3.0	85.0 ± 4.9	.04	0.54
Positional deformational plagiocephaly; number (%)	25 (19.2)	109 (66.1)	<.01	1.0
Positional brachycephaly; number (%)	112 (86.2)	128 (77.6)	.07	0.41

Data are presented as the median value (interquartile range).

Only cephalic index data is presented as the mean value ± standard deviation.

## 5. Discussion

### 5.1. Overall results

Here, we examined the cranial geometries of newborns during the early neonatal period at 2 Japanese facilities, during which growth-related parameters showed sex-related differences, but symmetry-related parameters did not. We were unable to identify any background factors that might predispose the patients to PDP. Symmetry-related parameters and the prevalence of PDP were substantially lower in the newborn group than in the 1-month-old group. This study is the first to quantify 3D cranial geometry parameters of Japanese newborns during the immediate postnatal period.

### 5.2. Measurement method

In this study, stereophotogrammetry was used to generate the 3D data files. Conventional handheld 3D scanners require head stability, making data collection from preterm or pre-neck-controlled infants challenging.^[19]^ Stereophotogrammetry can be used to construct 3D data by capturing images of an infant lying down and changing their neck directions using standard photographic techniques without specialized training to examine the cranial shape of preterm infants in the neonatal intensive care unit.^[9]^ These findings may aid future studies of cranial geometry in neonates and young infants.

The previously reported cranial shape data used for comparison^[6,8]^ were captured using a 3D scanner (Artec Eva, Artec, Inc., Luxembourg). Although we did not sufficiently evaluate reproducibility for the devices, the measurement method was unchanged, and we believe that there is no difference in the calculated data. Therefore, only the final measurement data were referenced.

Measurements were obtained at a mean of 3.3 days after birth, rather than immediately after birth. This delay allowed us to exclude early cranial changes (e.g., cranial deformation associated with delivery) and measure the infant’s natural cranial shape.

### 5.3. Growth-related parameters

Sex-based differences were observed in CC, CrW, cranial height, and CrV but not in CrL. The previous study was a longitudinal analysis of growth-related parameters among infants 1 to 6 months of age and identified similar trends among those at 1, 3, and 6 months of age.^[8]^ Although sex-based differences in body size, such as the CC and CrV, are well established, 3D measurements have clarified the lack of sex-based differences in CrL. The influence of factors such as birth canal shape was also considered. However, the exact cause remains unclear and should be investigated in future studies.

### 5.4. Brachycephaly

The mean CI in this study was 84%. Internationally, 86.2% of newborns exhibit PBC; in contrast, no newborn was classified as having PBC according to the Japanese criteria (CI > 93.9%).^[15]^ Japanese neonates tend to exhibit shorter heads immediately after birth than Western neonates.

Our initial hypotheses were as follows: vaginal delivery through the birth canal would result in an elongated cranium (indicated by a lower CI) than delivery by cesarean section, and the breech position would lead to a rounder head (indicated by a higher CI) than the cephalic position. However, our results contradict these hypotheses. Although the causes of this discrepancy remain speculative, it is possible that the effects were diminished because an average of 3 days had elapsed since delivery. Additionally, newborns severely affected by the delivery process may have been excluded from the study based on the criteria that eliminated those with bone intussusception and cephalohematoma. Furthermore, as only 8 participants were in the breech position, a larger sample size is needed to reexamine this issue in future research.

### 5.5. Plagiocephaly

The median CVAI score was low (2.1%). Similarly, the ASR and PSR, both volume ratios, exceeded 90% and were within normal ranges at birth, indicating that newborns exhibited minimal deformation immediately after birth. In a previous follow-up study of 1-month-old infants, the median CVAI was substantially higher, confirming the high incidence of cranial deformation in the Japanese population.^[6]^ Although the diagnostic threshold of PDP for CVAI was set at 5% in Japanese infants,^[4,6]^ this study found that Japanese newborns had a low incidence of cranial deformation immediately after birth, with only 9 (6.9%) exhibiting a CVAI > 5%. CVAI values also shifted to the left overall. Therefore, the diagnostic threshold for PDP in this study was set at a CVAI > 3.5% in accordance with international standards. Examination of background factors in patients with and without PDP revealed no notable risk factors. This trend was similar when cases were examined in patients grouped by a CVAI of >5%. Neonates that spent less time under the influence of gravity immediately after birth experienced fewer cranial deformations than those that did not.

Moreover, substantial differences in cranial deformities were observed in 1-month-old infants, suggesting that cranial deformation begins immediately after the infant is exposed to the effects of gravity outside the womb. These findings indicate that PDP is not due to background factors, such as pregnancy and delivery, but is strongly influenced by the postnatal environment.

### 5.6. Prospects

Takamatsu et al suggested that the prevalence of PDP is higher in Japan than in the West because of the traditional practice of raising children on their backs.^[4]^ In a study of 300 Japanese children up to 15 years of age, Akutsu et al reported a PDP prevalence of 46.7%, higher than that reported in Westerners.^[20]^ A previous report highlighted the importance of adequate care, such as tummy time, for infants whose skull shape worsens after 3 months of age.^[7]^ This study found that cranial deformation did not develop immediately after birth, indicating that controlling cranial shape is essential to preventing PDP. Thus, we propose a new hypothesis: it may be possible to reduce the incidence of PDP in Japanese infants to levels comparable to those in Western infants by providing sufficient education on cranial shape care immediately after birth and using devices designed to mitigate the effects of gravity on cranial deformation immediately after birth.

The United States Food and Drug Administration recommends prohibiting soft pillows because of their inability to prevent PDP.^[21]^ Therefore, new and safer devices must be developed to prevent future cases of cranial deformation.

### 5.7. Study limitations

A limitation of this study was the small number of participants, which might have compromised the subgroup analyses. Multivariate analysis of the risk factors for PDP was originally planned. With an estimated prevalence of PDP of 40% to 50% and 130 participants, we originally thought that 4 to 5 confounding factors could be considered (e.g., sex, fetal position, delivery method, and gestational age at birth). Contrary to our predictions, a multivariate analysis could not be performed because of the small number of patients with PDP. After dividing the CVAI values into 2 median groups and performing a logistic analysis of the above confounders (sex, fetal position, delivery method, and gestational age at birth) for the high- and low-CVAI groups, we found no significant risk factors. Due to the influence of sample size, the conclusions were at odds with clinical experience, particularly in the breech group. This raises questions such as whether this should be a simple sample size issue. Additional large-scale studies involving a larger number of deliveries, in collaboration with obstetric facilities, are required.

It was not possible to assess the results in relation to maternal physique or other factors. Furthermore, as the present study was limited to a single measurement immediately after birth, changes in cranial shape beyond the neonatal period should also be examined. Further large-scale studies are required in the future.

## 6. Conclusion

The cranial shape of newborns deforms after birth, and measures should be taken to prevent cranial deformities.

## Acknowledgments

We express our deepest gratitude to Berry Inc. for their assistance in analyzing the 3-dimensional data. No external funding was received for this study.

## Author contributions

**Conceptualization:** Yukari Tanaka, Hiroshi Miyabayashi, Nobuhiko Nagano, Ichiro Morioka.

**Data curation:** Yukari Tanaka, Hiroshi Miyabayashi, Yuri Kubota, Chihiro Mukai, Aya Nakanomori, Koichiro Hara, Katsuya Saito, Risa Kato.

**Formal analysis:** Yukari Tanaka, Nobuhiko Nagano, Ichiro Morioka.

**Investigation:** Hiroshi Miyabayashi, Ichiro Morioka.

**Methodology:** Hiroshi Miyabayashi, Nobuhiko Nagano, Ichiro Morioka.

**Project administration:** Hiroshi Miyabayashi, Ichiro Morioka.

**Resources:** Ichiro Morioka.

**Software:** Nobuhiko Nagano.

**Supervision:** Ichiro Morioka.

**Writing – original draft:** Yukari Tanaka, Hiroshi Miyabayashi.

**Writing – review & editing:** Yukari Tanaka, Hiroshi Miyabayashi, Nobuhiko Nagano.
